# Transmission experiments support clade-level differences in the transmission and pathogenicity of Cambodian influenza A/H5N1 viruses

**DOI:** 10.1080/22221751.2020.1792353

**Published:** 2020-07-23

**Authors:** Paul F. Horwood, Thomas Fabrizio, Srey Viseth Horm, Artem Metlin, Sopheaktra Ros, Songha Tok, Trushar Jeevan, Patrick Seiler, Phalla Y, Sareth Rith, Annika Suttie, Philippe Buchy, Erik A. Karlsson, Richard Webby, Philippe Dussart

**Affiliations:** a Virology Unit, Institut Pasteur du Cambodge, Institut Pasteur International Network, Phnom Penh, Cambodia; b College of Public Health, Medical and Veterinary Sciences, James Cook University, Townsville, Australia; c Department of Infectious Diseases, St. Jude Children’s Research Hospital, Memphis, TN, USA; d School of Applied and Biomedical Sciences, Federation University, Churchill, Australia; e GlaxoSmithKline Vaccines R&D Intercontinental, Singapore, Singapore

**Keywords:** Influenza, H5N1, avian, transmission, poultry, ducks, ferrets, Cambodia

## Abstract

Influenza A/H5N1 has circulated in Asia since 2003 and is now enzootic in many countries in that region. In Cambodia, the virus has circulated since 2004 and has intermittently infected humans. During this period, we have noted differences in the rate of infections in humans, potentially associated with the circulation of different viral clades. In particular, a reassortant clade 1.1.2 virus emerged in early 2013 and was associated with a dramatic increase in infections of humans (34 cases) until it was replaced by a clade 2.3.2.1c virus in early 2014. In contrast, only one infection of a human has been reported in the 6 years since the clade 2.3.2.1c virus became the dominant circulating virus. We selected three viruses to represent the main viral clades that have circulated in Cambodia (clade 1.1.2, clade 1.1.2 reassortant, and clade 2.3.2.1c), and we conducted experiments to assess the virulence and transmissibility of these viruses in avian (chicken, duck) and mammalian (ferret) models. Our results suggest that the clade 2.3.2.1c virus is more “avian-like,” with high virulence in both ducks and chickens, but there is no evidence of aerosol transmission of the virus from ducks to ferrets. In contrast, the two clade 1 viruses were less virulent in experimentally infected and contact ducks. However, evidence of chicken-to-ferret aerosol transmission was observed for both clade 1 viruses. The transmission experiments provide insights into clade-level differences that might explain the variation in A/H5N1 infections of humans observed in Cambodia and other settings.

## Introduction

The highly pathogenic avian influenza virus (HPAIV) A/H5N1 subtype has been a major, global public health concern since it was first detected in humans in Asia in 1997. In addition, novel subtypes such as the A/H5Nx, clade 2.3.4.4, and A/H7Nx viruses continue to emerge in the region [1,2]. Although zoonotic infection of humans is relatively rare, 861 cases of HPAI A/H5N1 in humans have been reported, with a case fatality rate of 53% [3]. Human-to-human spread of A/H5N1 has been suspected on several occasions [4], but the lack of sustained human-to-human transmissibility prevents A/H5N1 from spreading efficiently and reduces the risk of a potentially devastating pandemic. However, a human-transmissible strain of A/H5N1 might emerge in nature through the accumulation of mutations or via a reassortment event between A/H5N1 and a human influenza virus [5]. Indeed, experimental infections in ferret models have shown that the introduction of key substitutions in the hemagglutinin (HA) gene followed by serial passage of the virus can result in the accumulation of mutations that enable aerosol transmission of A/H5N1 between ferrets [6,7].

Influenza subtype A/H5N1 was first detected in Cambodian poultry in 2004, and it has since become enzootic in the country and continues to circulate at high levels in backyard farms and live bird markets [8]. Between 2004 and 2012, A/H5N1 viruses of clade 1 were the dominant circulating strains in Cambodian poultry: subclade 1 in 2004–2005; subclade 1.1 in 2006–2008; and subclade 1.1.2 in 2009–2012 [9–11]. In early 2013, a reassortant virus was detected circulating in Cambodian live bird markets. This virus contained the HA and neuraminidase (NA) genes from the previously circulating subclade 1.1.2 viruses and internal genes from clade 2.3.2.1a virus [10]. A dramatic increase in cases of A/H5N1 in humans in Cambodia coincided with the emergence of this reassortant strain, resulting in 26 cases in humans during 2013 and a further 8 cases in the first 3 months of 2014 [12]. The reassortant virus was subsequently replaced by clade 2.3.2.1c viruses in March 2014 and has not been detected since. Interestingly, since the introduction of the clade 2.3.2.1c virus to Cambodia, there has been only one reported case of A/H5N1 infection in a human in that country, despite consistently high levels of circulation of the virus in local poultry populations [8,13,14]. In the current study, we examined the avian and mammalian transmissibility of Cambodian A/H5N1 isolates in an effort to account for the increase in cases in humans during 2013–2014, which was associated with the circulation of the clade 1.1.2 reassortant virus, and the curious lack of cases in humans since the introduction of the clade 2.3.2.1c virus.

## Materials and methods

### Viral strains

Three Cambodian influenza A/H5N1 isolates were used in this study to represent the three dominant strains that have circulated in that country since 2009: (1) A/Cambodia/W0526301/2012 (clade 1.1.2); (2) A/Cambodia/X0817302/2013 (clade 1.1.2 reassortant, with clade 2.3.2.1a internal genes); and (3) A/Duck/Cambodia/Y0210303/2014 (clade 2.3.2.1c). The same viral isolates were used in the experiments undertaken at the Institute Pasteur in Cambodia (IPC) (Phnom Penh, Cambodia) and at St. Jude Children's Research Hospital (St. Jude) (Memphis, Tennessee, USA). The full genomes of the clade 1 viruses (W0526301 and X0817302) included in this study have been previously published [10]. The genome of the clade 2.3.2.1c virus has also been previously published [15]. The accession numbers and other relevant information for all three viruses are included in Supplementary Table 1.

### Ethics statement

For the studies performed at the IPC, all procedures were approved by the National Ethics Committee in accordance with international regulations on Animal Care and Use. For the studies performed at St. Jude, all procedures were approved by the St. Jude Children's Research Hospital Institutional Biosafety Committee (IBC) and Animal Care and Use Committee (IACUC) and were in compliance with the Guide for the Care and Use of Laboratory Animals. These guidelines were established by the Institute of Laboratory Animal Resources and were approved by the Governing Board of the US National Research Council.

### Laboratory facilities

All infection experiments at both facilities were conducted in Biosafety Level 3 enhanced containment laboratories. Investigators were required to wear appropriate protective equipment and to follow all necessary precautions in accordance with enhanced biosafety. Ducks and chickens were housed in HEPA-filtered, negative-pressure, animal isolators. For chicken-to-ferret transmission studies, all animals were housed in open cages with food and water ad libitum. The cages were separated by approximately 15 cm and were contained within a flexible film isolator (Class Biologically Clean, Ltd.) maintained under constant negative pressure with HEPA-filtered supply and exhaust.

### Duck-to-duck transmission

For each study virus, three 6-week-old mallard ducks (*Anas platyrhynchos*) (donors) were inoculated with 10^4^ 50% chicken lethal infectious doses (LD_50_) of A/H5N1 in a volume of 200 μL via intranasal inoculation. Four hours post inoculation, six uninfected 6-week-old mallard ducks (contacts) were placed together with individual infected donors in animal isolators. All birds were monitored daily for clinical signs of disease, morbidity, and mortality. Oropharyngeal and rectal swabs were collected from all animals daily, and serum samples were collected on days –3, 0, 1, 3, 7, 10, 15, 20, 25, and 28 post contact. Organ samples (from trachea, lung, air sac, liver, spleen, kidney, heart, intestine, brain, muscle, and feathers) were collected from each animal upon death or at day 28 post contact.

Nucleic acids were extracted from all swabs and organ samples by using the QIAamp Viral RNA Mini Kit (Qiagen, Hilden, Germany) in accordance with the manufacturer's instructions. Extracts were screened for influenza A matrix (M) gene RNA by using real-time RT-qPCR protocols from the International Reagent Resource (https://www.internationalreagentresource.org/Home.aspx). Viral RNA levels were quantified using serial dilutions of an influenza A/H5N1 standard, enumerated by plaque assay.

Avian serum samples were screened for anti-A/H5N1 antibodies by hemagglutination inhibition (HI) assays, using 0.5% chicken red blood cells and performed according to previously described protocols [16,17]. Briefly, influenza A/H5N1 viral antigens from each of the study viruses were standardized to 4 HA units/25 µL. The HI titration end-point was defined as the reciprocal of the highest dilution of virus that caused complete hemagglutination. All birds were also screened for anti–influenza A antibodies by HI assay 3 days before the experiments commenced to rule out previous exposure to the virus.

### Duck-to-chicken transmission

Each study virus was intranasally inoculated into three 6-week-old mallard ducks (donors), using the protocols outlined above. Four hours post inoculation, six uninfected 6-week-old chickens (*Gallus gallus*, native Cambodian chickens) (contacts) were placed in animal isolators with individual infected ducks. The experimental protocol was performed as outlined in the duck-to-duck transmission experiments described above.

### Chicken-to-ferret transmission

For each study virus, three 3- to 4-month-old ferrets (Triple F Farms, Sayre PA) (donors) were intranasally inoculated with 10^4^ chicken LD_50_ of A/H5N1 in a volume of 200 μL. Twenty-four hours after inoculation (1 d post infection [dpi]), one ferret from each virus group was individually cohoused with a single influenza-naïve ferret (to mimic contact transmission) and placed alongside another influenza-naïve animal that shared the same airspace but was separated from the other ferret by a double-layer wire mesh to prevent direct interaction (and thereby permit only airborne transmission).

To further test the zoonotic potential of the viruses, 10^4^ 50% chicken LD_50_ of A/H5N1 of each study virus, in a volume of 200 μL, was inoculated into three white leghorn chickens (*Gallus gallus domesticus*) via the natural route. After 24 h, four naïve chickens were placed in the cage with each inoculated chicken (to mimic contact transmission) and a cage of three naïve ferrets was placed at a distance of approximately 0.3 m from a cage of white leghorn chickens (to mimic conditions permitting airborne zoonotic transmission). For both sets of experiments, nasal washes were taken from each ferret at 1, 3, 5, 7, and 9 dpi. Oropharyngeal and cloacal swabs were collected from all chickens at 1, 3, 5, and 7 dpi or until the terminal endpoint, and serum samples were collected from ferrets at 20 or 21 dpi. Infectious virus titres were determined for each nasal wash and oropharyngeal swab by 50% tissue culture infectious dose (TCID_50_) assays, using limiting dilution on MDCK cells and the calculation method of Reed and Muench [18]. HI assays were conducted as described above to determine whether ferrets had seroconverted.

## Results

### A/H5N1 viruses transmit efficiently between poultry

For data analysis, the poultry were allocated to one of three groups for each of the study viruses: experimentally infected donor ducks (*n* = 6, including three ducks infected in the duck-to-duck experiments and three ducks infected in the duck-to-chicken experiments), contact ducks (*n* = 6), and contact chickens (*n* = 6).

**Figure 1.**
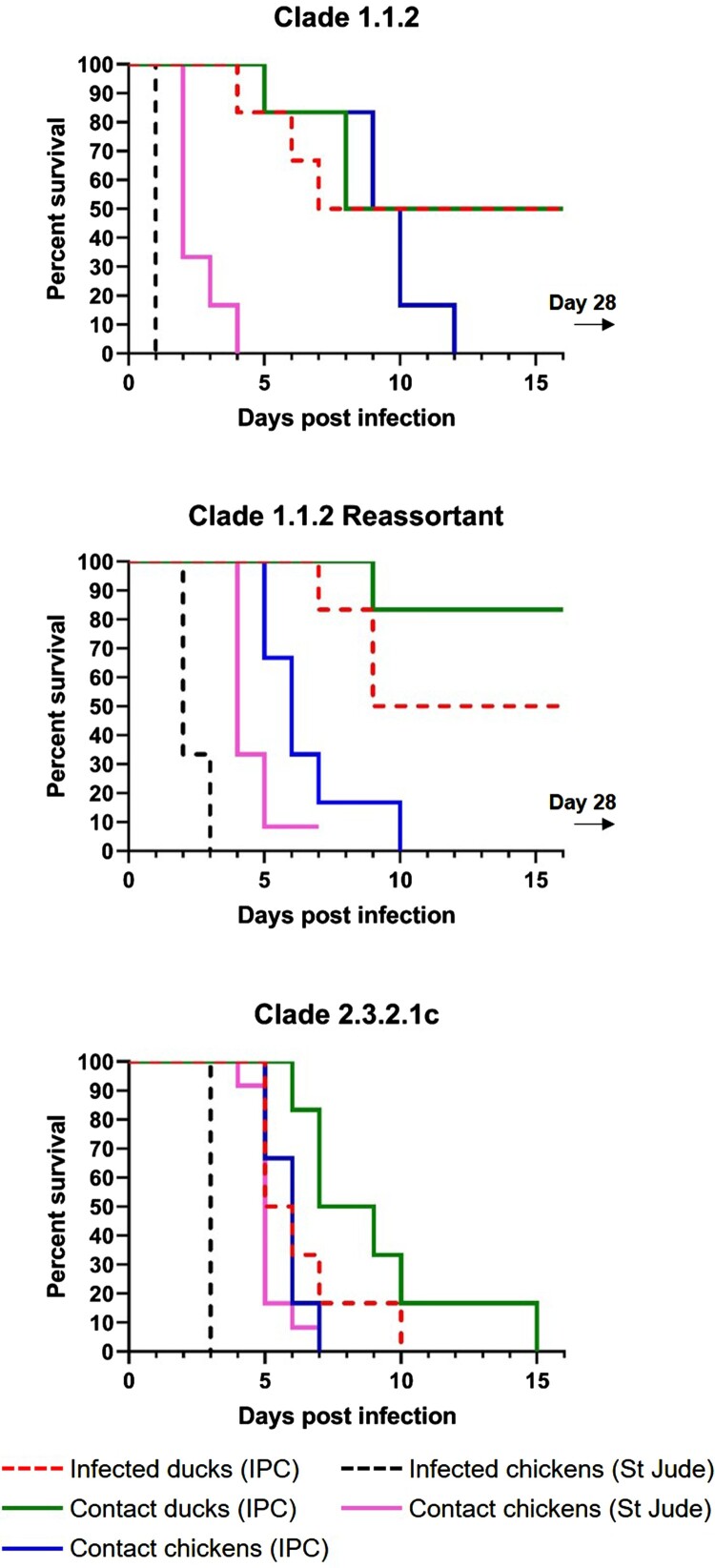
Poultry survival curves comparing three influenza A/H5N1 viral clades. IPC: Institute Pasteur in Cambodia; St Jude: St. Jude Children’s Research Hospital.

Contact exposure of chickens resulted in 100% mortality with all three A/H5N1 viruses tested. For contact chickens exposed to clade 1.1.2, clade 1.1.2 reassortant, or clade 2.3.2.1c virus, the mean time to mortality was 8.2, 5.5, and 4.8 days, respectively. Experimental and contact exposure to the clade 2.3.2.1c virus resulted in 100% mortality in ducks. In comparison, the clade 1.1.2 virus caused 50% mortality in both experimentally infected ducks and contact ducks, and the clade 1.1.2 reassortant virus caused 50% and 16.8% mortality in experimentally infected ducks and contact ducks, respectively (Figure 1). For ducks infected with or exposed to clade 1.1.2, clade 1.1.2 reassortant, or clade 2.3.2.1c virus, the mean time to mortality for experimentally infected ducks was 5.7 (*n* = 3/6), 8.3 (*n* = 3/6), and 6.3 (*n* = 6/6) days, respectively, and that for contact ducks was 7 (*n* = 3/6), 9 (*n* = 1/6), and 9 (*n* = 6/6) days, respectively. In contrast to the outcomes in ducks, contact exposure of chickens resulted in 100% mortality with all three A/H5N1 viruses tested. The mean time to mortality for contact chickens exposed to clade 1.1.2, clade 1.1.2 reassortant, or clade 2.3.2.1c virus was 9.2 (*n* = 6/6), 6.5 (*n* = 6/6), and 5.8 (*n* = 6/6) days, respectively.

**Figure 2.**
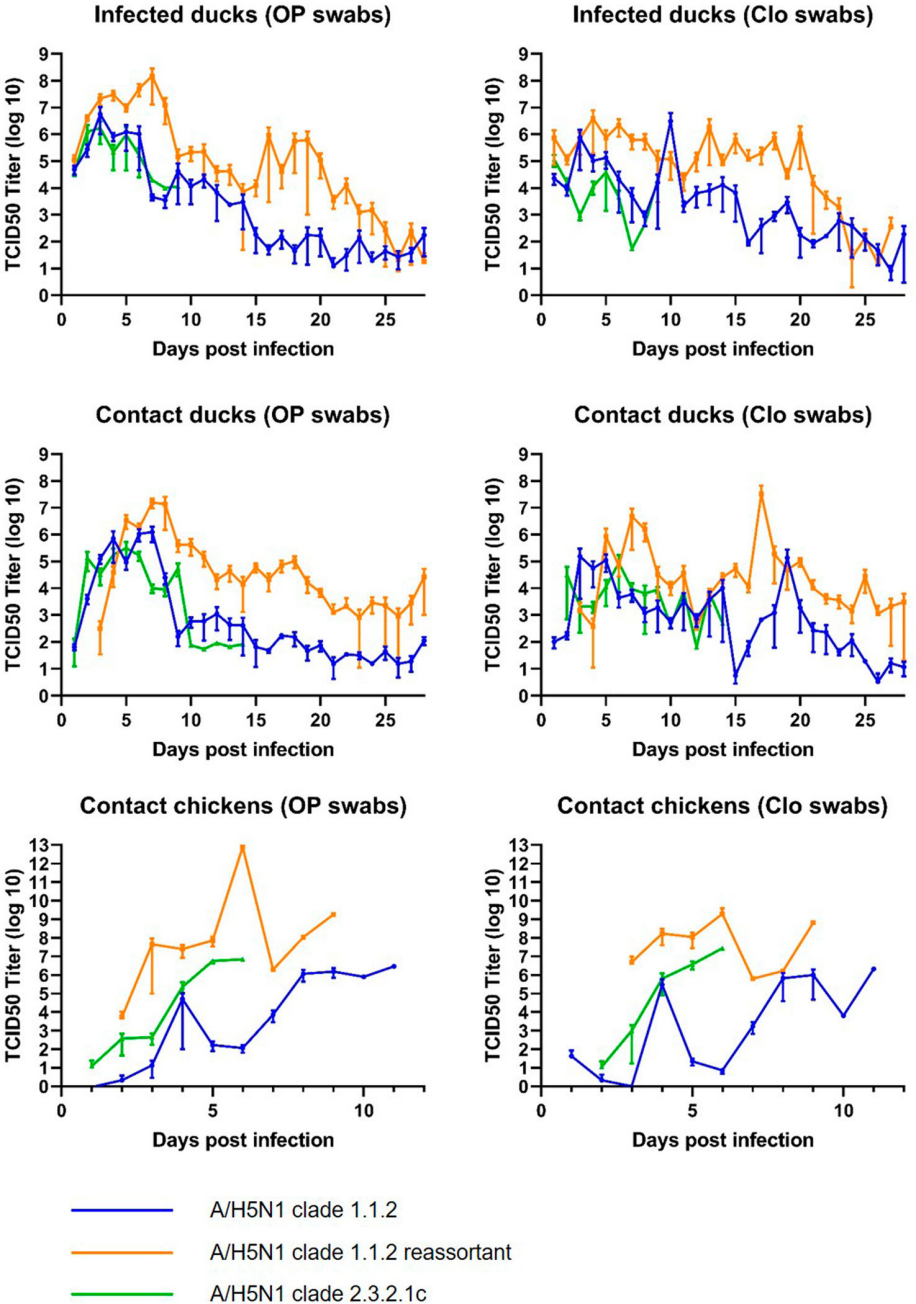
Detection of influenza A(H5N1) RNA in oropharyngeal (OP) and cloacal (Clo) swabs of experimentally infected ducks, contact ducks, and contact chickens.

Viral shedding, as determined by the detection of viral RNA by qRT-PCR, was consistently higher in ducks and chickens infected with the clade 1.1.2 reassortant virus than in birds infected with either of the other two viruses tested (Figure 2). Persistent shedding of viral RNA was also detected in ducks infected with either of the clade 1 viruses, with high levels of virus still being detected in the oropharyngeal and cloacal swabs of some animals at day 28. Persistent shedding from ducks infected with the clade 2.3.2.1c virus could not be assessed because all experimentally infected ducks died by day 9 and all contact ducks died by day 14. All three viruses caused systemic infections in all experimental animals, and persistent infection of multiple internal organs was detected in animals throughout the study (Figure S1). All ducks had positive seroconversion to the challenge virus (Figure S2); however, seroconversion was not detected in any of the chickens, presumably because they died before antibodies could be detected. Viral levels were universally high in all organs in contact chickens.

### Contact transmission of A/H5N1 viruses from poultry to ferrets

The chicken data analysis was performed using three groups of chickens, with five chickens for each study virus (*n* = 15 chickens in total). Each virus was inoculated into three chickens, with four naïve contact chickens being provided for each inoculated chicken. Additionally, there was one naïve aerosol contact ferret for each group of five chickens per test virus (*n* = 3 ferrets in total).

**Figure 3.**
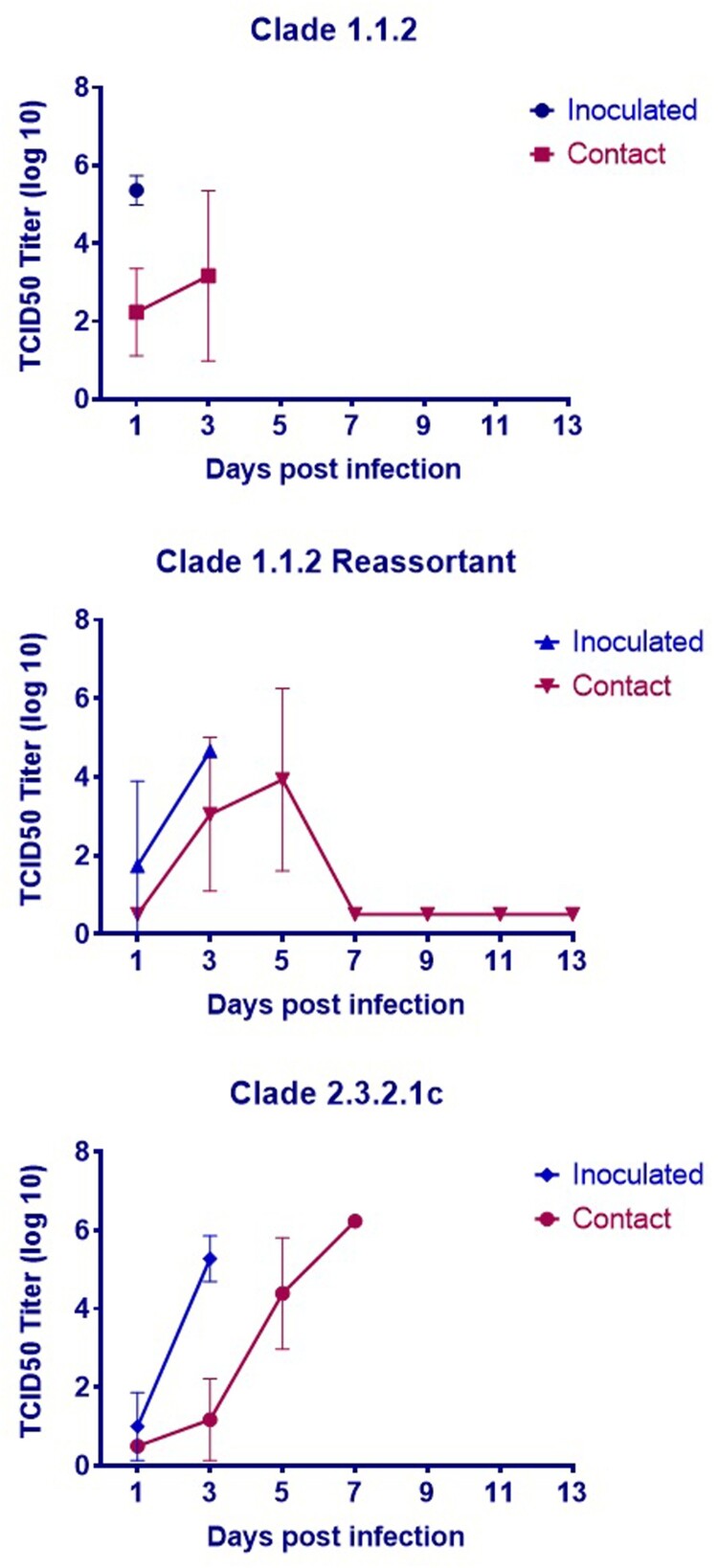
Chicken viral titres in oropharyngeal and cloacal swabs during transmission experiments.

**Figure 4.**
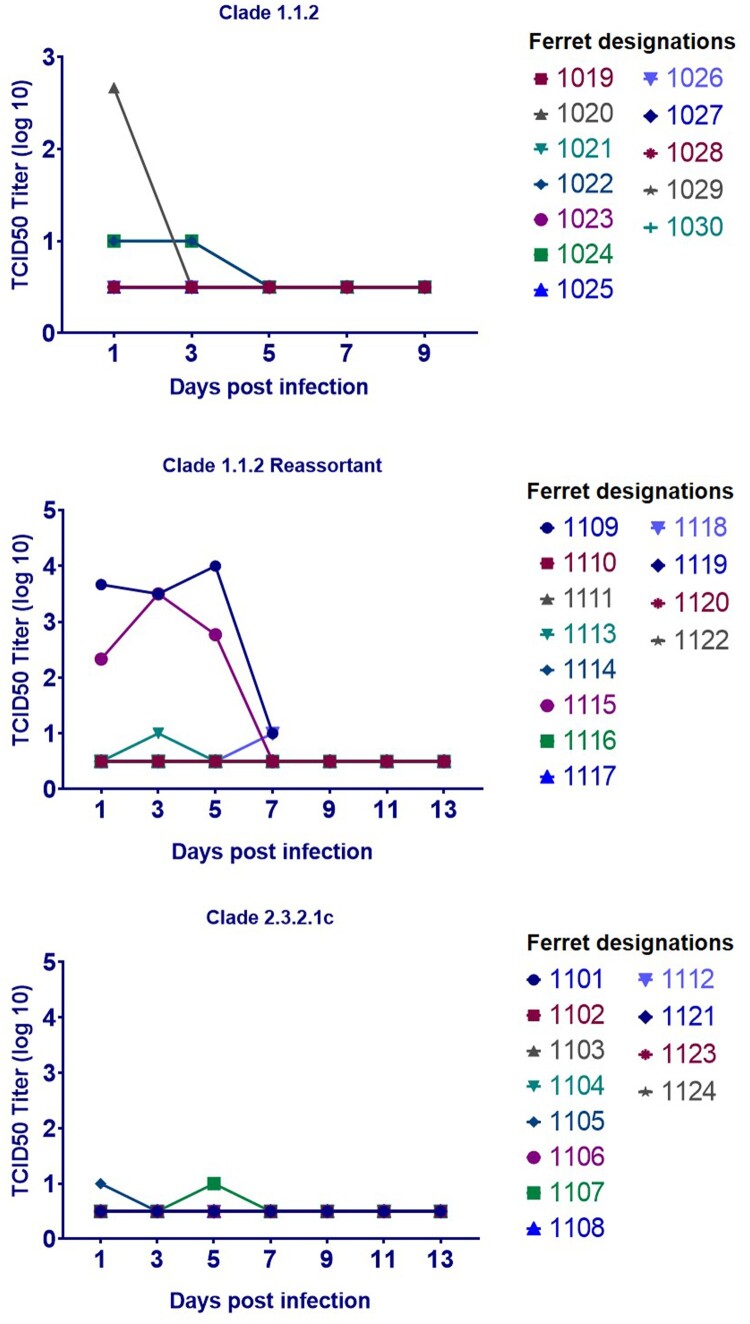
Ferret viral titres detected from nasal washes.

All three viruses were lethal in chickens within 3 days of inoculation, and all contact chickens exposed to the clade 1.1.2 or clade 2.3.2.1c virus succumbed to the infection (Figure 1). Only one contact chicken survived in the clade 1.1.2 reassortant virus group, and no virus was detected in the oropharyngeal or cloacal swabs of this animal at any time point (Figure 3). Although there were no detectable viral titres in nasal washes collected from chicken aerosol contact ferrets (data not shown), two of the chicken aerosol contact ferrets in the clade 1.1.2 virus group (#1028 and #1029) and one of the ferrets in the chicken aerosol group for the clade 1.1.2 reassortant virus (#1118) had to be euthanized after developing severe neurologic signs (Figure 4), possibly associated with systemic influenza infection. Additionally, one of three directly inoculated ferrets (#1109) in the clade 1.1.2 reassortant virus group also succumbed to the infection. Interestingly, this occurred at 8 dpi and after detectable viral titres in the nasal wash had decreased to the limit of detection. No viral titres were detected in the nasal washes of any of the direct contact (ferret–ferret) or aerosol contact ferrets for any of the three test viruses (Figure 4).

Similar to the ferret-to-ferret experiment, chicken aerosol exposed and inoculated ferrets in the clade 1.1.2 and clade 1.1.2 reassortant virus groups exhibited weight loss and intermittent increase in body temperature indicative of influenza infection, but no virus was detected (Figure S3). The ferrets with the most weight loss were the ones euthanized.

To determine whether the zoonotic transmission of the clade 1.1.2 and clade 1.1.2 reassortant viruses was significant, the experiments were repeated with the same animal arrangements. As expected, all chickens succumbed to the infections in 2–4 dpi while shedding large amounts of virus, as detected by both the oropharyngeal and cloacal swabs, and there was no detectable virus in the nasal washes of all three ferrets in each test virus group (Figure S4). However, in contrast to the first experiments, no clinical signs were observed in chicken aerosol contact ferrets during the repeat experiments.

### Clade 1.1.2 virus infection in ferrets results in systemic spread without detectable viral shedding in nasal washes

**Figure 5.**
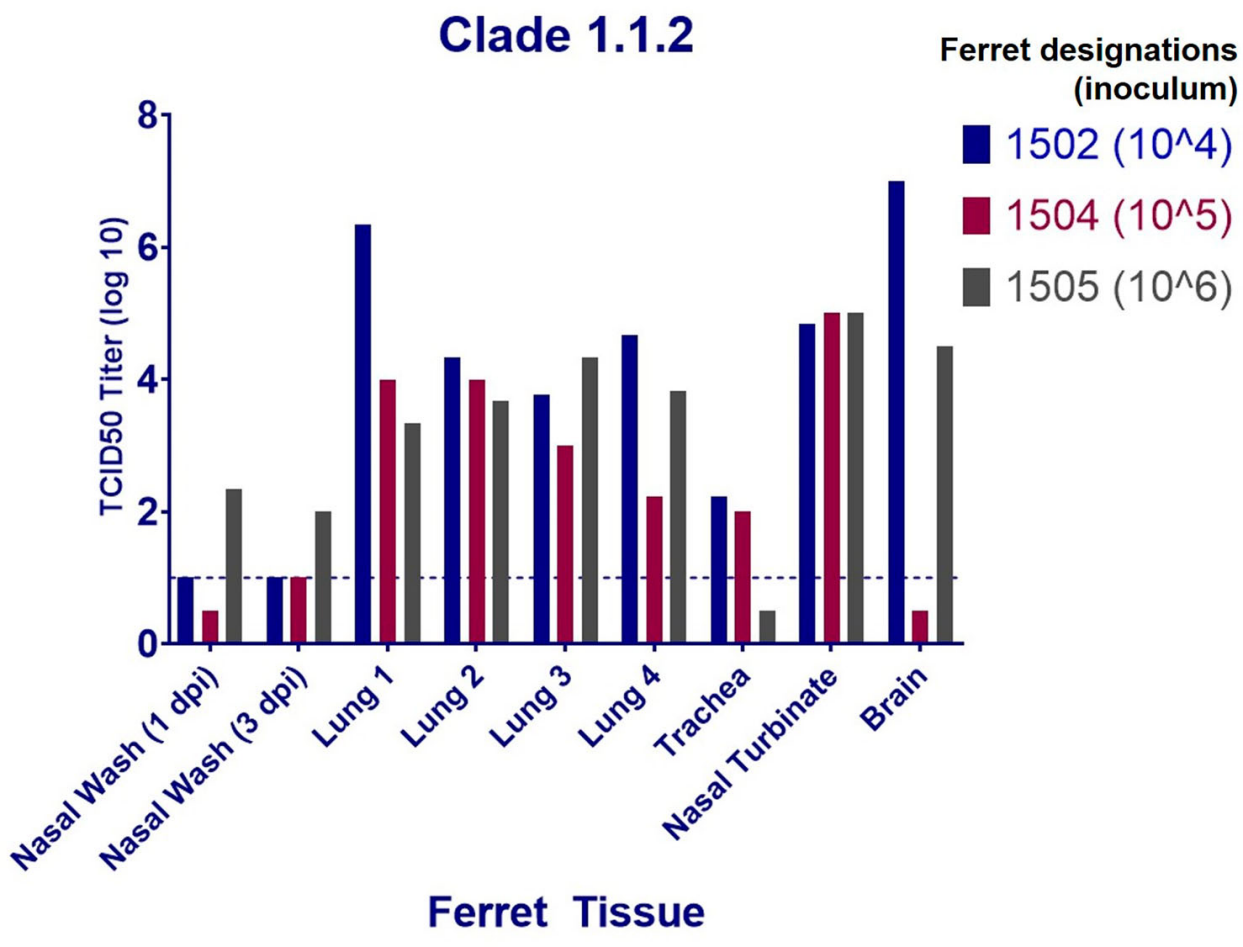
Viral titres detected in the organs of ferrets infected with three different doses of the A (H5N1) clade 1.1.2 virus.

Exposure to the clade 1.1.2 virus resulted in severe clinical signs in ferrets exposed to infected chickens but there was no detectable virus in their nasal washes; therefore, we wanted to assess whether this virus was capable of systemic spread without nasal shedding. To test this, three ferrets were inoculated with three different viral titrations ranging from 10^4^ to 10^6^ TCID_50_/mL and nasal washes were collected at 1 and 3 dpi followed by tissue collection at 4 dpi. Nasal washes from the ferret inoculated with 10^6^ TCID_50_/ml had detectable viral titres at both 1 and 3 dpi; however, the titre was low and appeared to decrease from 1 dpi to 3 dpi. The two ferrets inoculated with 10^4^ and 10^5^ TCID_50_, respectively, had nasal wash viral titres at or below the limit of detection at both 1 and 3 dpi (Figure 5). Interestingly, despite the low viral load detected in the nasal washes, virus titres were relatively high in the nasal turbinates of all of the animals, as well as in four individual lung lobes and in the brains of two of the three ferrets, despite the different inoculum titres (Figure 5).

## Discussion

Avian influenza A/H5N1 viruses of the Goose/Guandong/96-lineage have spread to more than 70 countries around the globe and are now considered enzootic in domestic birds in China, Southeast Asia, the Middle East, and Africa [19]. Over time, this avian influenza virus lineage has evolved to form 10 recognized clades and many more subclades [20], all of which can be traced back to the original HA gene isolated from a goose in Southern China in 1996 [21]. Some of these clades have displayed fitness for sustained circulation in poultry, whereas others have quickly disappeared from circulation. In addition, it appears that some viral clades have displayed a greater ability to jump the species gap and infect humans [22]. Despite the apparent differences between A/H5N1 clades and subclades with respect to their fitness in poultry and mammals, few *in vivo* studies have been conducted to compare directly the virulence and transmission of these different groups of viruses.

In the present study, the clade 1 viruses displayed lower virulence in ducks and greater poultry-to-mammal transmissibility than did the clade 2.3.2.1c virus. These characteristics are consistent with viruses that have a greater pandemic risk, which is supported by the greater number of infections of humans by the clade 1 viruses, as compared to clade 2.3.2.1 viruses, reported in Cambodia and globally [22]. Poultry-to-mammal aerosol transmission was suspected with both of the clade 1 viruses. Severe neurological illness was observed in two ferrets exposed to clade 1.1.2 virus–infected chickens and in one ferret exposed to clade 1.1.2 reassortant virus–infected chickens (in addition to one ferret directly infected with the clade 1.1.2 reassortant virus). However, A/H5N1 virus was not detected in the nasal washes from any of these animals. A repeat of this experiment with clade 1 viruses did not result in clinical signs suggestive of A/H5N1 infection or in any detectable virus in the ferret nasal washes, suggesting that zoonotic transmission of these viruses requires specific conditions and is not a frequent occurrence. There was no contact or aerosol transmission of the clade 1 viruses between ferrets. This is similar to what is seen in nature, where the avian virus can cross the species barrier but cannot transmit further. However, relatively high levels of virus were detected in the nasal washes of ferrets experimentally infected with the clade 1.1.2 reassortant virus. Increased levels of upper respiratory tract replication could be an indication of greater affinity for mammalian cell receptors [23]. The greater shedding of the reassortant virus from infected ducks and chickens, coupled with the relatively high levels of virus in the nasal turbinates of infected ferrets and evidence of intermittent aerosol transmission of the virus between poultry and mammals, indicates that further *in vitro* and *in vivo* experiments are necessary to derive an in-depth virological risk assessment of the Cambodian clade 1.1.2 reassortant viruses. Genomic analyses conducted in previous studies [10] and in the present study (Supplementary Table 1) did not reveal any mutations that may have been responsible for the increased risk of mammalian infection with the clade 1.1.2 reassortant virus.

Clade 2.3.2.1 viruses first emerged in 2008 [24] and have since spread and diversified to become one of the dominant lineages of A/H5N1 viruses globally. The clade 2.3.2.1c lineage was first detected in Cambodia in February 2014, replacing the previously circulating clade 1.1.2 reassortant virus to become the only influenza A/H5N1 virus circulating in that country and remaining so up to the present (March 2020) [8,10]. A/H5N1 virus infections in humans have dramatically decreased (with only one confirmed case) since the introduction of this virus into Cambodia. Meanwhile, live bird market surveillance and poultry outbreak investigations have established that high levels of clade 2.3.2.1c viruses have circulated in poultry populations during this time [14], suggesting that this virus is less able than its predecessors to jump the species gap into humans. Global data appear to support this hypothesis, with there being few reports of clade 2.3.2.1c virus infections in humans, despite widespread poultry circulation in China, Indonesia, Vietnam, and West Africa [25–29]. The frequent detection of clade 2.3.2.1 viruses in wild birds and their rapid global dissemination [24,30] may indicate that these viruses are more “avian-like,” affecting their ability to infect humans. Further studies are needed to investigate the *in vivo* characteristics of these viruses and the possible genetic mechanisms associated with their host specificity. Indeed, clade 2.3.2.1 viruses have been significantly associated with wild birds, suggesting that inherent characteristics of that virus subclade may enhance its ability to establish infection and spread in some species in the natural reservoir population. In the present study, the clade 2.3.2.1c virus was considerably more virulent in the duck species used in this experiment than were either of the clade 1 viruses, resulting in mortality of all experimentally infected and contact ducks within 9 days, except for one contact duck that died on day 14. Previous studies have also provided evidence that clade 2 viruses have evolved to be more pathogenic in ducks, by comparison with their clade 1 ancestors [31], with unusually high mortality rates in duck flocks being reported during outbreaks [25,32].

The greater virulence of clade 2.3.2.1c viruses in avian hosts may have come at the cost of less efficient transmissibility to mammalian hosts. The clade 2.3.2.1c virus did not cause illness in ferrets directly infected with the virus or in ferrets subjected to aerosol contact with infected chickens. However, Pearce et al. [33] described a clade 2.3.2.1c duck isolate from Vietnam (Dk/VN/0004) that was highly pathogenic in ferrets, suggesting that within-clade genetic differences also play an important role in pathogenicity, transmission, and host specificity. The authors compared the sequence of Dk/VN/0004 with another clade 2.3.2.1c isolate that was non-pathogenic in ferrets and although they noted a number of amino acid differences (8 in the polymerase genes and 3 in the HA gene) only one (T188I) has been associated with increased binding to mammalian receptors [34]. The recent emergence of clade 2.2.1.2 viruses causing many cases in humans in Egypt over a short period [35] and the evolution of reassortant H5Nx viruses from the 2.3.4.4 lineage [36] show that the perceived lower pandemic risk from clade 2 viruses is not universal and that close monitoring of these viruses is still needed.

Our findings suggest that some A/H5N1 viruses can establish persistent infections in ducks. Clade 1 viruses were detected in multiple organs of all of the ducks euthanized at day 28 (Supplementary Figure 1), and cloacal and respiratory shedding were also consistently detected (Figure 2). Hulse-Post et al. [37] found that clade 1 viruses (isolated in 1997–2004) were persistently shed by ducks for up to 17 dpi. Wibawa et al. [38] also reported persistent detection of viral RNA up to 24 dpi in ducks experimentally infected with a clade 2.1.1 virus, but infectivity could not be confirmed beyond 10 dpi. Persistent shedding might increase the transmission of these viruses to humans through greater contamination of the environment. Persistent infection of organs, with the very high levels of virus concentration detected in the present study, poses a risk for poultry slaughterers and for people preparing birds for cooking.

In this study, we observed differences in viral shedding and virulence in Cambodian native chickens (*Gallus gallus*) and leghorns. There is a need for further validation of these differences. In particular, further studies are needed to investigate the pathogenicity and transmission of A/H5N1 and other avian influenza viruses in native poultry. To date, most avian influenza transmission studies have been conducted in developed countries (where the necessary facilities exist), using avian species that may not accurately represent the bird species that are common in enzootic countries. Various studies have documented that individual viruses can have highly variable pathogenicity in a range of bird species [39–41].

A limitation of our study was that only one representative virus from each clade was included in the transmission experiments. Within-clade variation in transmission and virulence is likely and has been reported previously in transmission experiments [30]. In addition, the two clade 1 viruses were both isolated from cases of A/H5N1 infection in humans, whereas the clade 2.3.2.1c virus was isolated from a poultry outbreak associated with the death of approximately 4500 ducks [42]. The prior replication of the clade 1 viruses in a mammalian (human) host could conceivably have influenced the transmissibility of these viruses between avian and mammalian hosts, as compared to that of the avian clade 2.3.2.1c virus. Only one clade 2.3.2.1c virus infection of a human has been detected in Cambodia to date and, unfortunately, no virus could be isolated from that patient. Despite this possible limitation, none of the viruses included in the experiments contained critical mutations (e.g. Q222L and G224S) associated with mammalian adaptation. Therefore it is likely that the viruses selected for these experiments reflect the three main clades of A/H5N1 that have circulated in Cambodia and their risk of transmission to humans.

In this study, we showed differences in avian virulence and avian-to-mammalian transmission between three successive A/H5N1 clades that have circulated in Cambodia since 2009. The transmission experiments provided insights into the dramatic increase in cases in humans after the emergence of the clade 1.1.2 reassortant virus, along with the subsequent paucity of cases following the replacement of this virus by a clade 2.3.2.1c virus. These patterns are consistent with observations from the field, where a considerable reduction in cases of A/H5N1 virus infection in humans has been observed in all countries where clade 2.3.2.1c viruses are the dominant circulating clade, despite high levels of ongoing circulation in domestic poultry populations. Aerosol transmission of the two clade 1 viruses from poultry to ferrets provided further support for the hypothesis that these viruses were more likely to cause human infections than was the clade 2.3.2.1c virus. In addition, persistent infections of multiple organs and high levels of virus shedding were observed in all ducks that survived the infection with clade 1 viruses. Further studies are needed to validate these findings and to elucidate the mechanism of persistence, as this may have implications for the risk of human exposure and the spread of A/H5N1 through the poultry trade and migratory birds.

## Supplementary Material

12_-_Supp_table_1_-_virus_information__PH121018_.xlsx

11_-_Figure_S4_-_St_Judes_1.1.2_and_1.1.2R__010720_.jpg

10_-_Figure_S3_-_St_Judes_ferret_weight_and_temp.jpg

09_-_Figure_S2_-_serology__010720_.jpg

08_-_Figure_S1_-_Organs__190320v2_.jpg
