# Supplementary figures and images for: Transmission experiments support clade-level differences in the transmission and pathogenicity of Cambodian influenza A/H5N1 viruses
Source: Emerg Microbes Infect. 2020 Jul 23;9(1):1702–11. doi: 10.1080/22221751.2020.1792353 (PMC7473085; doi:10.1080/22221751.2020.1792353)

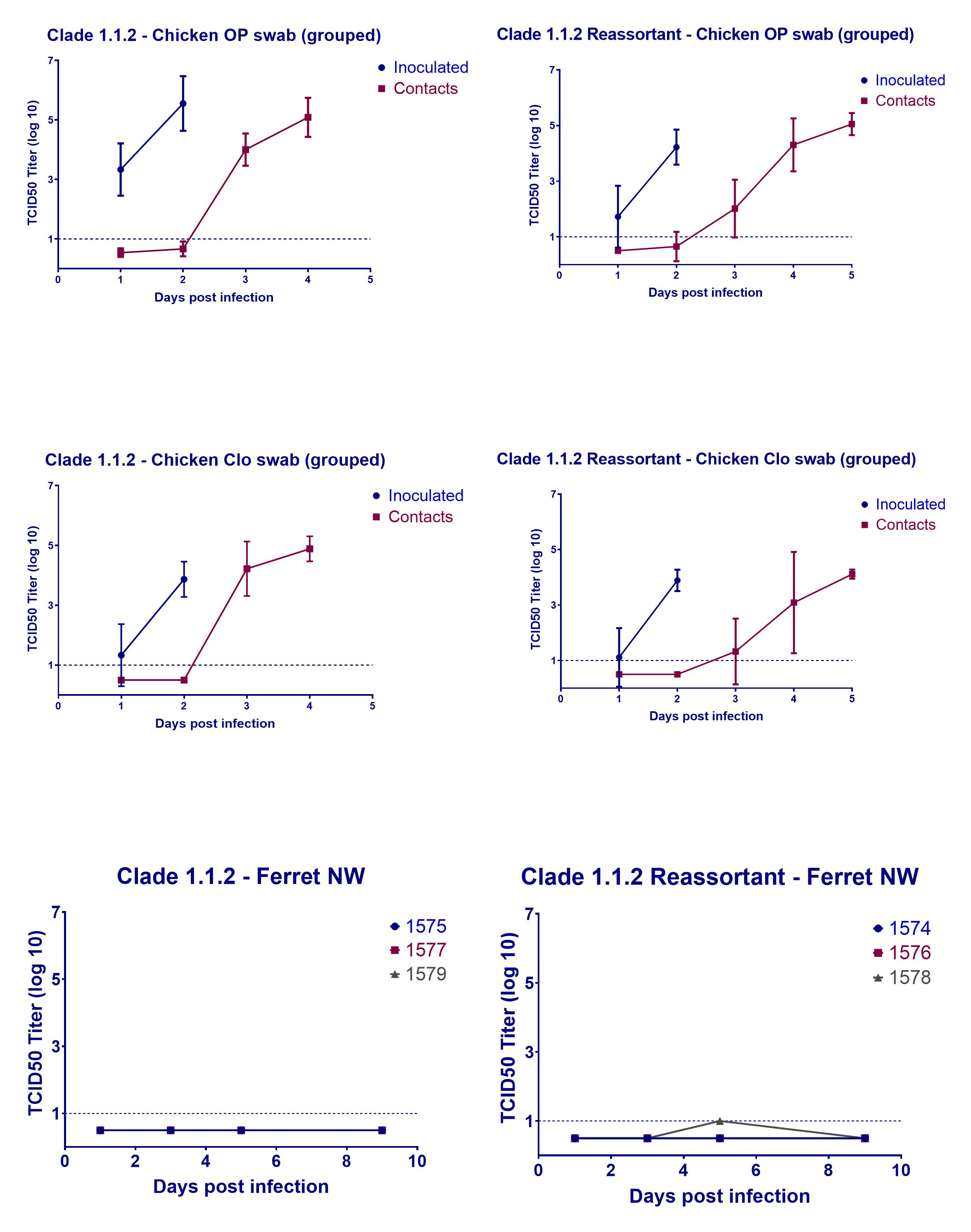

Supplement: 11_-_Figure_S4_-_St_Judes_1.1.2_and_1.1.2R__010720_.jpg [file TEMI_A_1792353_SM9738.jpg]

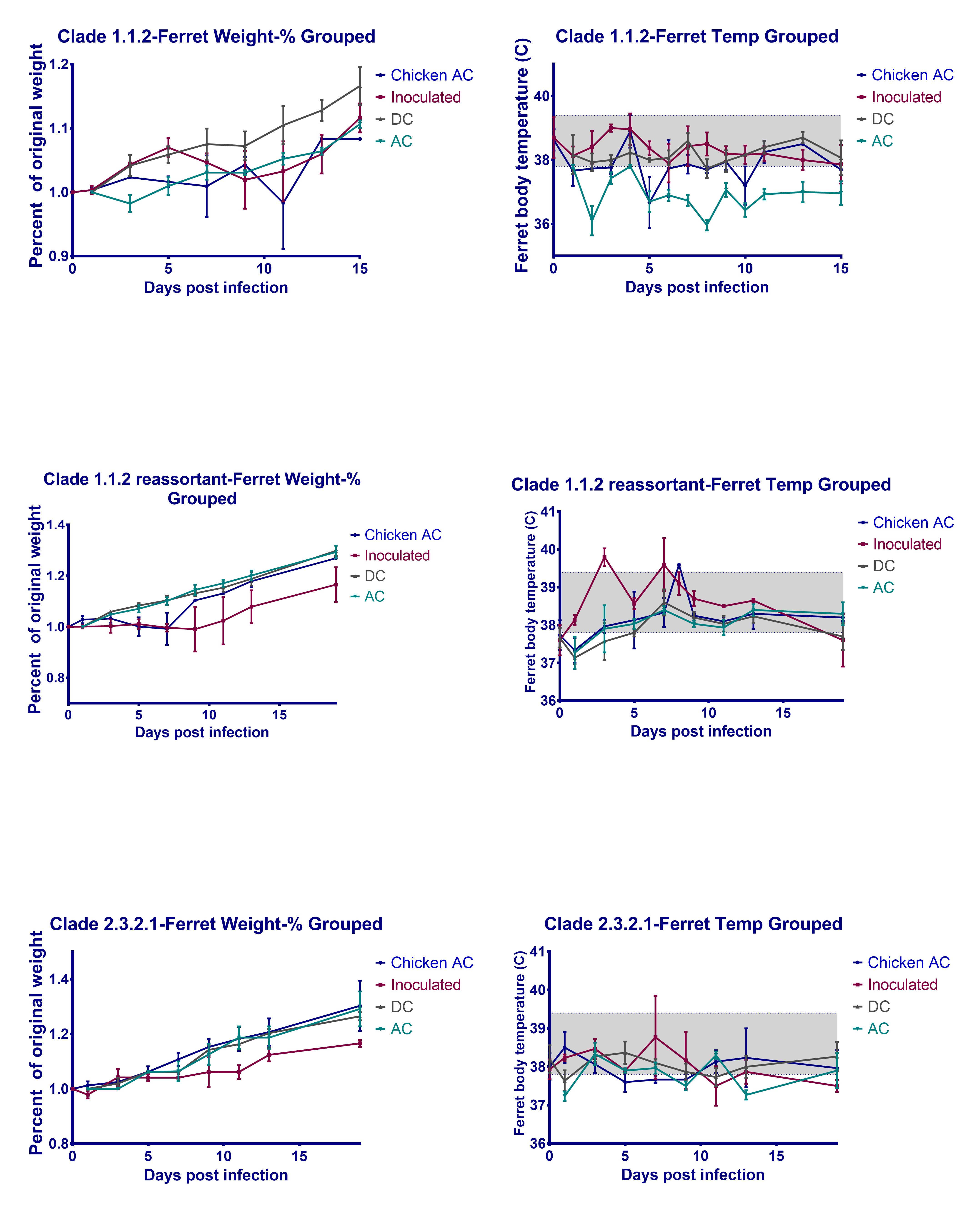

Supplement: 10_-_Figure_S3_-_St_Judes_ferret_weight_and_temp.jpg [file TEMI_A_1792353_SM9737.jpg]

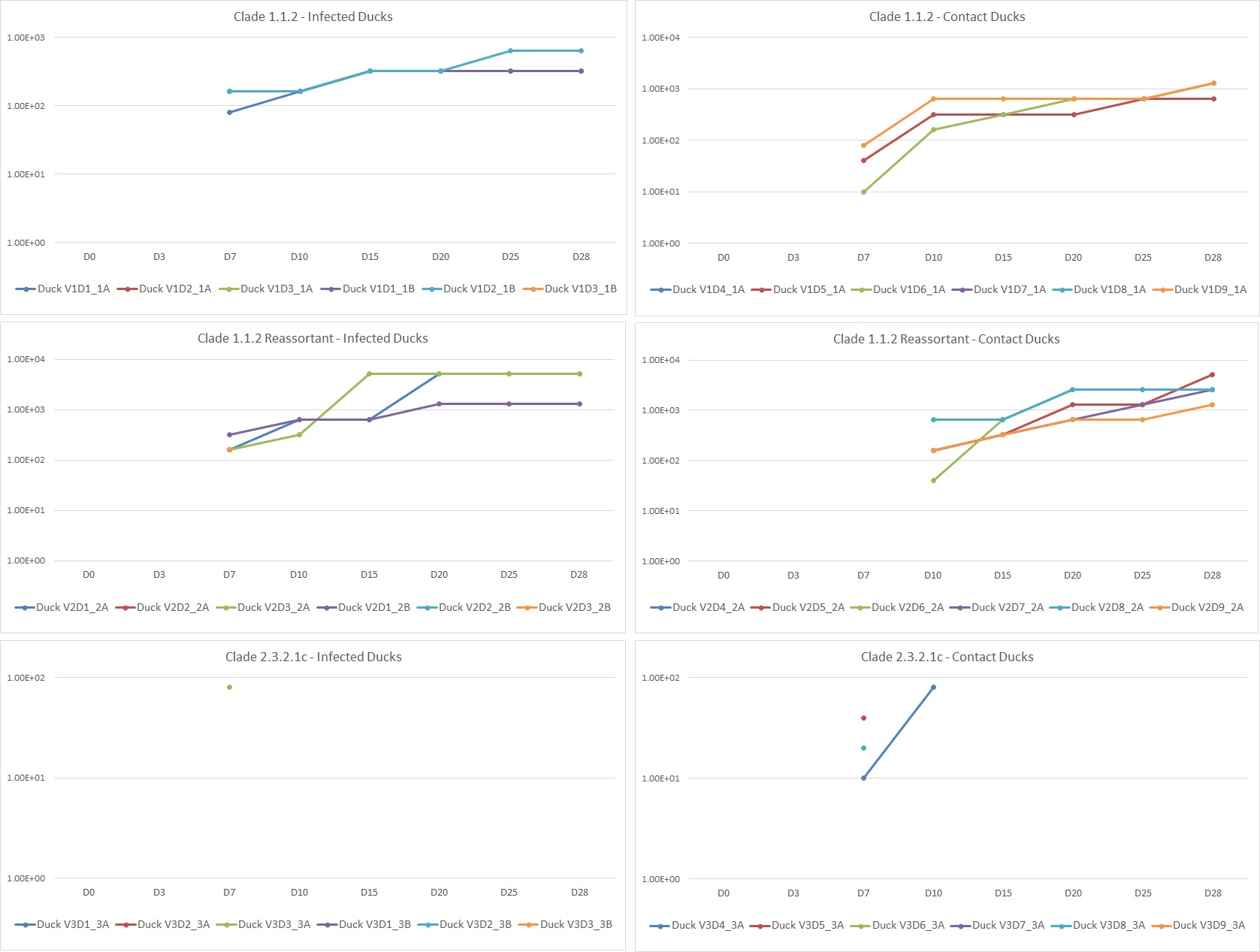

Supplement: 09_-_Figure_S2_-_serology__010720_.jpg [file TEMI_A_1792353_SM9736.jpg]

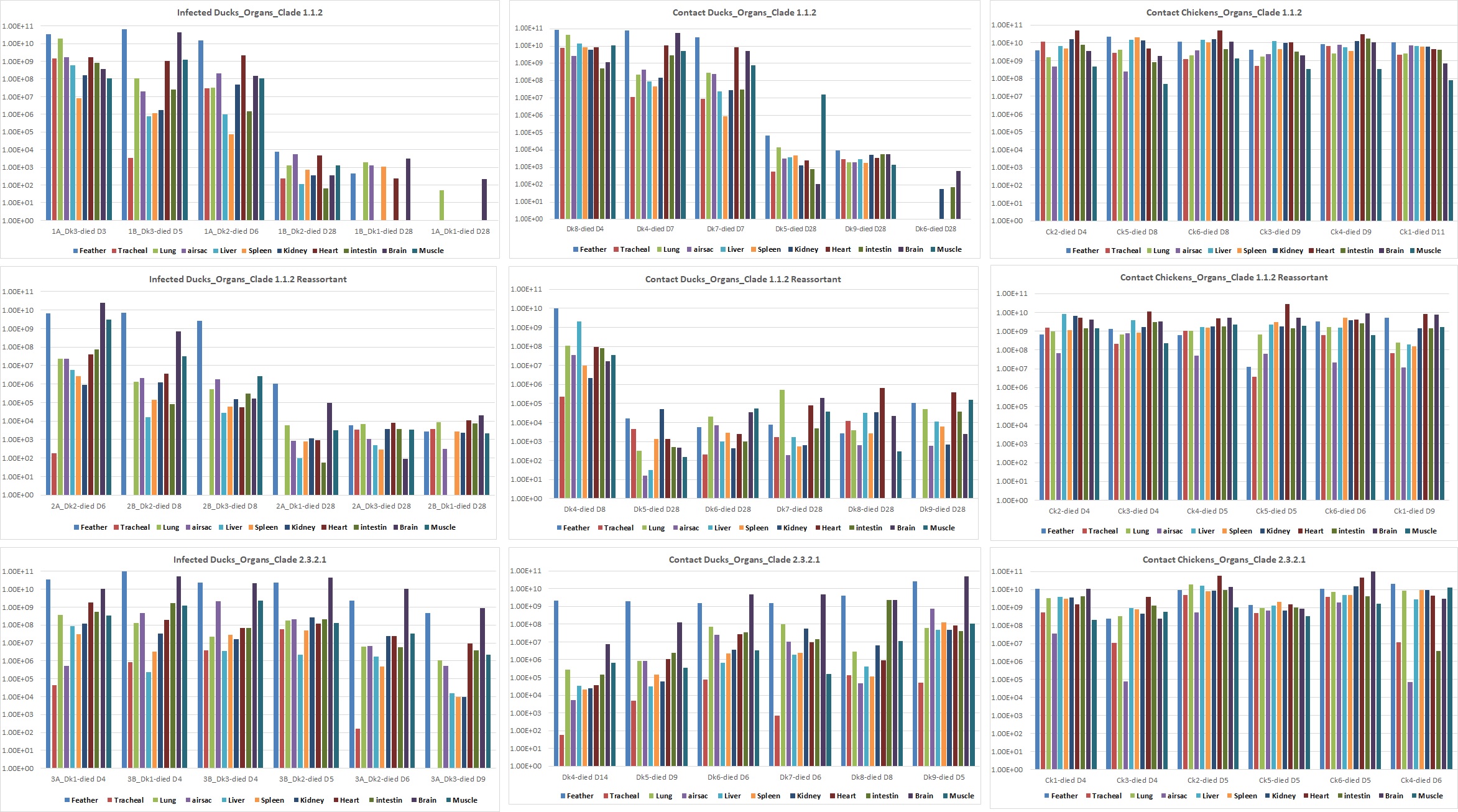

Supplement: 08_-_Figure_S1_-_Organs__190320v2_.jpg [file TEMI_A_1792353_SM9735.jpg]
